# Notes of an European Tour

**Published:** 1846-10

**Authors:** F. H. Hamilton

**Affiliations:** Professor of Surgery in Geneva Medical College


### BUFFALO MEDICAL JOURNAL

AND

### MONTHLY REVIEW.

VOL. 2. OCTOBER, 1846. NO. 5.

### ORIGINAL COMMUNICATIONS.

ART. I.—*Notes of an European Tour.* By F. H. HAMILTON, M. D. *Professor of Surgery in Geneva Medical College.*

I shall in this be accused of incorrect taste, at least by all antique-worshipers, but still I venture to affirm that Powers' Eve, and Greek slave, are superior to the Venus de Canova, or the Venus de Medici. I do not judge either of them by academic rules, for in these I am wholly uninstructed; my judgment is based solely on my notions of feminine beauty and character, in both of which the two works of the American artist excel, as well indeed as in anatomical accuracy and exquisite delicacy of finish. Before the Venus de Medici and the Venus de Canova, I feel disappointment mingled with admiration; before the Eve and Greek slave I feel only admiration mingled with tears. I cannot look upon these Divine creations without emotion — Mr. Powers has hewn out for himself immortality.

Mr. Powers himself accompanied me to the orthopædic establishment of Prof. Carbonai, nearly opposite his studio in the Via della Furnace. The establlshment is very large, having in all 135 rooms, including a gymnasium, a school room, chapel, dining rooms, operating rooms, bed rooms, bathing rooms, etc. Within a court, also is a neat and refreshing little garden. He has now about 50 patients. I counted 30 inclined planes and beds of Procrustes, variously and ingeniously constructed for pulling and straightening the spine. Upon these the patients are exercised occasionally, but not retained long at a time. All the apparatus worn for lateral curvatures, is so constructed as to admit of free exercise of all the muscles. Talipes he treats by section of tendons and by apparatus, and I have only to accuse him of quackery in his assertion that he cures varus, 3d degree, in two months! Whoever asserts this, whether of New-York, Philadelphia, Paris, or Florence, asserts a falsehood. A varus of 3d degree may be straightened often in one minute, but it is never *cured* in two months, nor in three, as the experience of every honest and practical surgeon must testify.

Prof. Carbonai showed me a host of casts as trophies, but I have very little confidence in plaster or in pictures, either of which can more easily be made straight, than crooked feet. Do not think I have lost faith in tenotomy as applied to clubbed feet. I know and appreciate its value, but the profession have been deceived and been led at last to undervalue it, from the declarations of dishonest and interested men that cures were accomplished *speedily*, when the truth is that long and patient perseverence is almost always necessary.

This establishment has been in operation now 5 years, the Prof. receiving a salary directly from the Grand Duke, and giving daily lectures to a small class of medical students. I think that no where in Italy is the science of medicine and surgery, at present, so highly cultivated as in Florence. And, indeed, the University of Florence is the only one in Italy whose medical department can boast a respectable number of pupils. Why should it not be so? The Tuscan dominions are better governed, I think, than any other portion of Italy; its Dukes are more liberal; its lands are better cultivated; its peasantry and all the poor are less poor and more intelligent; its principal cities, Leghorn and Florence, are more thriving. Leghorn is made a free port, and they have *dared* to build a railroad to Pisa. In short, the country presents every where, evidences of *civilization*; while most of Italy seems scarcely to have emerged from barbarism. Florence was the cradle of the fine arts under the Medici — the Medici — do you know that this family of Merchant Princes and the patrons of the arts and sciences, were originally poor Doctors, and hence were called "Medici": and to this day the old palaces which they once occupied are recognised by the three huge balls or pills over the door, their ancient profession being thus honorably respected in their quarterings. But I was about to say that Florence also sustained such men as Steno and Bellini, both of whom were physicians to Cosmo III — as Mascagni the anatomist, and Nobile the naturalist, and Micheli "the lynx of botany," and Galileo; the body of the latter lies in the church of Santa Croce, but his *dried finger* you may see in the tribune of the museum of Natural History! I have not spoken of Dante, Bocaccio, Petrarca, Corilla, Michæl Angelo, and Machiavelli, who lived and flourished at Florence, nor of Americo Vespucci, who was born where the hospital di St. Giovanni di Dio now stands, all of whose names have added lustre to the fame of this city, and as they promoted science and art generally, so they have contributed to sustain the science of medicine. And thus it is that at present Prof. Buffalini, who lectures in the University, is probably the most distinguished physician in Italy, and Prof. Andreini the most skillful surgeon, and that to Florence the eyes of all Italy are directed as the center of Italian medical literature.

Pisa, the once great city and republic, whose chains you see at Genoa, is now a small, deserted, sorrowful looking town, and has little worthy to be seen beyond its famous cemetery, called Campo Santo, its barbaric, or as I would say, its fancy-patch-work cathedral, baptistry, and leaning tower, or campanile. The latter is a sort of cork-screw belfry, eight stories high and built of the finest marble, black and white — a second tower of Babel; erected solely to hang therein the church bells. When I had counted its 290 steps, I leaned against the iron rail of the upper terrace quite exhausted, and thought how much better these things were ordered at Bunker Hill, where one is pulled up in a bucket for a shilling! But what can be expected of a people who perhaps never heard of a wooden clock, and never dreamed how a Yankee would have saved to Æneas the *labor* and *opus* of his long walk up from Avernus, by an inclined plane and stationary engine.

The University of Pisa was founded in 1339, and has numbered among its Professors, Andrew Cæsalpinus, who, although he did not trace out the complete vascular circulation, was able, as early as 1571, to describe correctly the course of the blood through the heart, and the use of the valves. Fallopius (from whom the fallopian tubes were improperly named, since they were fully described before his time;) was Prof. of Anatomy at Pisa in 1548, and Mercuriali, (physician, knight and count palatine,) in 1599. Here also labored but lately, A. Vacca Berlinghieri, the great Pisan surgeon, and who endeavored with M. Sanson, to introduce the recto-vesical cutting for stone.

The University building, a plain structure, accommodates now in all its departments about 500 students. Its botanical garden was laid out in the year 1595, during the most palmy days of the institution, and it still continues a considerable source of attraction to the scientific traveller. The degree of M. D. is conferred at the University after four years study, but the new doctor is not allowed to practice until he has received two additional years of clinical instruction, and been subjected to a new examination.

At Leghorn, I stopped at the excellent hotel, kept by Mr. Thompson, an Englishman. Mr. Thompson says Leghorn is no place for a consumptive patient, nor is Pisa much better. The winds are variable, often cold and damp. And few who come to this part of Italy suffering under confirmed pthisis ever return to their families. Only two weeks since, Mr. C. B., a young lawyer from Cincinnati, Ohio, died at this house. He had come to Italy by advice of his friends, confident of a complete restoration, if ever he could reach Pisa. He had spent the winter at Pisa. but his malady, consumption, rapidly progressed, and about a month before he died, he came to Leghorn. After his death, the rooms which he had occupied were repainted and thoroughly whitewashed, and all the clothes as carefully cleaned and aired as if he had died of small pox. This Mr. Thompson was compelled to do in deference to the opinions held by Italians of the contagiousness of consumption. During the last winter an American was received into an Italian hotel in this city, and as soon as the landlord learned that he had consumption, he compelled him to be moved out of his house, and with his exceedingly low condition, and the mental suffering at being driven into the streets, he died while they were carrying him down stairs!

The morning I left Leghorn, a heavy wind was blowing in from the sea, and the French government steamer, for which I had been waiting, dared not attempt an entrance into the harbor. Seeing her at anchor about two miles out, and fearing that she would determine to proceed without coming in, Doctor Wilson, sen. physician to Leeds Hospital, and myself threw ourselves into a crazy yawl, managed by a couple of stout boatmen, whom we had selected from a whole crew of applicants, as looking best able to contend with a rough sea. But I think now that had I seen fully before I left the quay, the gambols old Neptune was that day playing, I should scarcely have trusted myself in his dominions. After nearing the steamer, we found no little difficulty in keeping ourselves from being thrown under her wheels by the violence of the waves; and when at length we got on board, we presented a most pitiable appearance, my companion having lost his hat in the melee, and both of us being as thoroughly drenched as if we had been at the bottom of the sea. All the night and the following day, the wind continued to blow almost a hurricane, and the next night at about 11 o'clock, when near Marseilles, we run into a schooner with a tremendous crash, her bow striking one of our paddles, and carrying away much of the bulwarks on that side, besides maiming us seriously in other respects. The shrieks and cries for help which immediately came from the schooner, continuing until they were lost by the distance, led us to suppose that she was sinking. But our Captain kept coolly on his way in spite of the remonstrance of his passengers, and what was the fate of the vessel and her crew, I shall propably never know.

At Marseilles, I visited the Hospital of Hotel Dieu, a civil and military establishment, which was founded in the 12th century. It is large, but situated in the old and a very crowded part of the city, so that its ventillation is imperfect. In one portion of the building are the fever patients, in a second, venereal cases, in a third, surgical, and in a fourth, the invalid soldiers. There is also a small ward devoted exclusively to protestants. Here I saw a young American lad just recovering from a fever.

The whole number of patients is about 400. With this establishment is connected one of the French secondary or preparatory schools of medicine, consisting of eight chairs and five adjunctships, all filled by very able men.

From Marseilles to Avignon we came by post, and from thence to Lyons by the Rhone, I found on board the steamer, Dr. Carbonai, brother of the orthopœdist, of Florence, and Dr. ———, one of the physicians to the Grand Duke of Tuscany : they are en route for Paris and London, to visit the hospitals, &c. Dr. Carbonai enquired whether Americans spoke English, and added by way of compensation for the information which I gave him, that Niagara Falls "etait detruit"—saying that the rock had given way, and left only a long inclined plane! This he affirmed upon the authority of the *Herald* which he had seen in a reading-room in Florence, and which paper may be seen in almost every reading-room on the Continent, from Amsterdam to Naples. The Herald is our principal representative abroad.

But a fine looking old lady from Grenoble, who had been down to Marseilles to see her son, was also exceedingly inquisitive. I was not very well, and had stretched myself at length on some cushions, and ordered a plate of strawberries, when the old lady began to question me after this manner: "Are you sick, sir?" "Where are you going?" "Where are you from?" "America! but you are white, and Americans are *black!*" "Have you parents?" "Have you strawberries in America?" "Have you steamboats?" "Railroads?" My replies to the last inquiries, "ten times as many as in France," made her look quite incredulous. But she seemed soon to recover her confidence in my honesty, and finally urged me with great earnestness, to come to Grenoble and spend a week or more at her house. "It is," said she, "the handsomest city in the world." She had never seen Paris. All this time she was holding in her lap a kitten, which she kept constantly kissing and petting and stroking, and who was as constantly biting her fingers, and snarling like any spoiled child. I thought I might ask her also, one question. "Are you very fond of cats?" "Very," said she, "I have ten or twelve at home!"

"Hotel Dieu," at Lyons, is near the river, on a fine, open street, and its principal facade looks like a palace. Some of the salles extend through the whole length of the building, and the beds are all of them supplied with light curtains. Every thing is perfectly clean, the windows are large, the nurses attentive, and altogether, I have been more favorably impressed with its appearance than with any hospital on the Continent. M. Petrequin is Surgeon-in-Chief, and M. Bouchacourt "Aid Major"—the latter is also Surgeon-in-Chief to "La Charite." I saw nothing but straight splints on fractured thighs, done up in well padded junks.

"La Charite" is a rich "poor-house," with about one thousand inmates. It contains a large work-shop also, the machinery in which, is carried by a steam engine. In the washing room were eight or ten girls at work, one of whom my guide addressed as having the general supervision of this department. She was a beautiful girl, of eighteen or twenty, dressed in the costume of a nun, and is the daughter of one of the wealthiest families in the city. Her sleeves were rolled up nearly to her shoulders, and her arms were as deep in the suds as any of the paupers about her.

Adjoining the salle, which is devoted to the foundlings, is a small receiving room, in which one of the nuns sits night and day; and at all times a small fire is kept in a stove which heats a "creche" or crib, where the little outcasts are placed until they become sufficiently warm to wash and dress them. The receiving wheel, is a kind of revolving shelf, or rather, three shelves in a half cylinder. The children are placed on one of these shelves which are kept facing the street, and being then turned by the harlot mother without, a bell is made to ring and the nun takes the child. Over the mantle-piece stands a clock, and in a large book is recorded the year, day, hour and minute of the reception, the sex of the child, its condition as to apparel, appearance, health, &c. Along one side of the wall hang small ivory medals, each inscribed with a numeral. One of these medals is suspended upon the neck of the infant; if a male, the medal bears the additional inscription E. G., if a female, E. F. The number being entered in the record, serves as the name by which the wearer of the medal may hereafter be identified with the description, and by which the mother, if she chooses, may at any period recognize and reclaim her child.

Near the summit of the lofty hill, upon the almost perpendicular sides of which much of the old town is built, stands the "Hopital de l'Antiquaille," or the ancient hospital, in which the insane are confined. It looks without like a prison of the middle ages. Having no "permit" I could not enter.

Signor Carbonai tells me there is an excellent orthopoedic establishment in the city. Andry, who wrote in the beginning of the eighteenth century, on "the art of preventing and removing deformities in children," was born at Lyons. Lyons was also the cradle of Bichat's studies. In the secondary medical school established here, you will recognize the names of Braschet, the therapeutist, and whose excellent treatise on opium in certain inflammations, ought to be read by every student and practitioner of medicine; of Janson, the surgical pathologist; of Dupasquier, the pharmaceutist, and of Bonnet, the distinguished surgeon, all of whom have made valuable contributions to medical and surgical science. It was in Lyons, also, that Levrat acquired his distinction as a surgeon.

From Lyons to Chalons sur Saone, we had a delightful ride on the steamer "Aigle." Yet there was very little approaoh to the speed of an "eagle" in our ascent of the river, having grounded twice, and seldom making over six miles an hour. From Chalons to Paris, we resumed the old time-honored conveyance in which a Frenchman loves to travel — the diligence.

